# Supplementary material for: Association between oral microbiome and seven types of cancers in East Asian population: a two-sample Mendelian randomization analysis
Source: Front Mol Biosci. 2023 Nov 21;10:1327893. doi: 10.3389/fmolb.2023.1327893 (PMC10702768; doi:10.3389/fmolb.2023.1327893)
Supplement: Supplementary file 1 [file DataSheet1.ZIP › Supplementary Materials/MR plots for tongue/tongue═╝/Lung cancer/pheno.2004_to_lung cancer_forest.pdf]

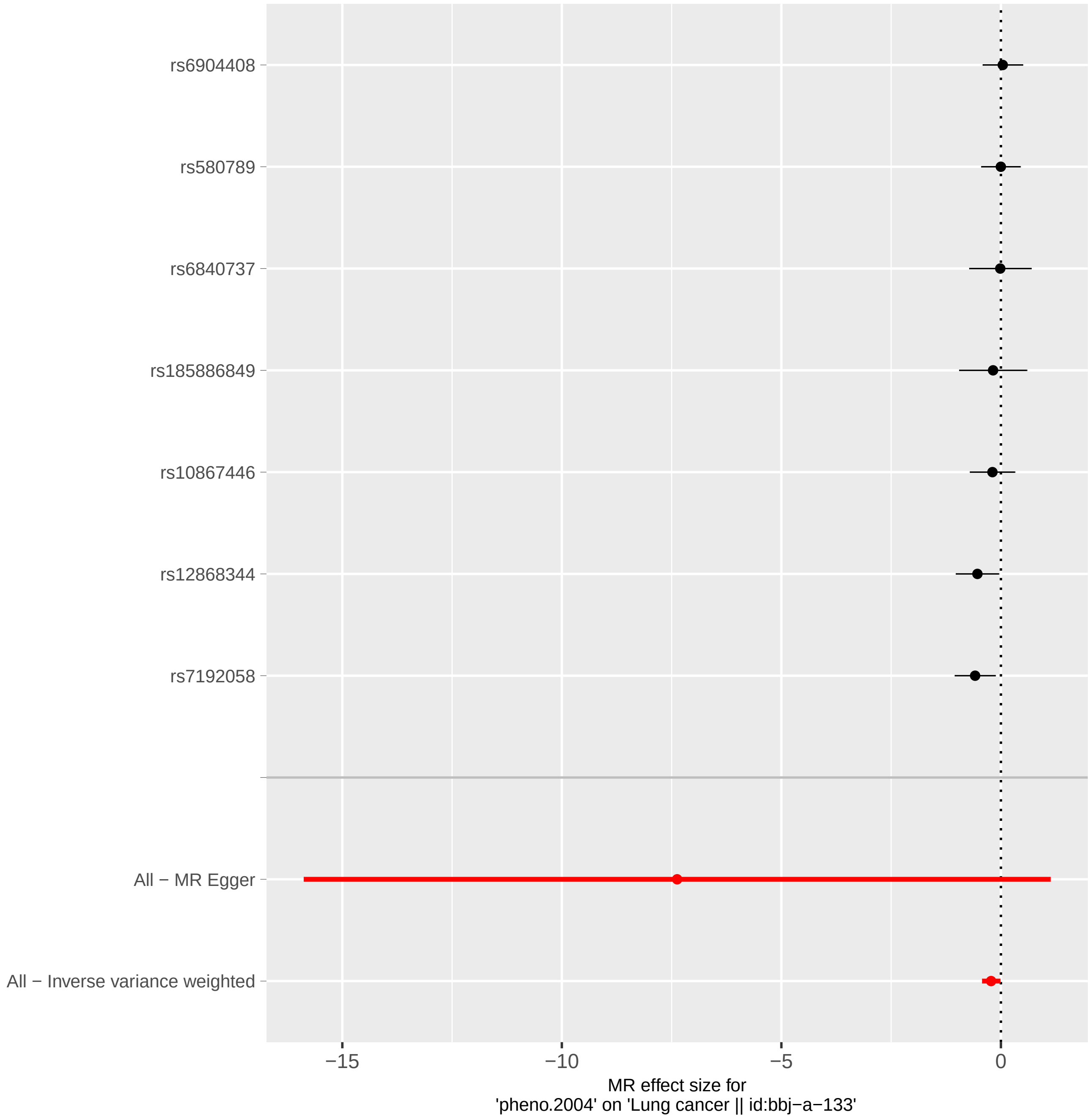
rs6904408
rs580789
rs6840737
rs185886849
rs10867446
rs12868344
rs7192058
All − MR Egger
All − Inverse variance weighted
−15
−10
−5
0
MR effect size for
'pheno.2004' on 'Lung cancer || id:bbj−a−133'
